# Red cell distribution width to albumin ratio (RAR) as a potential biomarker of coronary heart disease (CHD): Insights from a cross-sectional study

**DOI:** 10.1097/MD.0000000000046663

**Published:** 2025-12-19

**Authors:** Rong Lei, Huiling Liang, Xumeng Ding, Chaofu Yue, Xian Huang, Qiaolin Li, Wei Bao, Qi Qiu, Mei Yang

**Affiliations:** a Department of Intensive Care Unit, Yunnan Qujing Central Hospital (Qujing No. 1 People’s Hospital), Qujing, Yunnan, China; b Medical Administration Office, Yunnan Qujing Central Hospital (Qujing No. 1 People’s Hospital), Qujing, Yunnan, China; c Department of Cardiology, Yunnan Qujing Central Hospital (Qujing No. 1 People’s Hospital), Qujing, Yunnan, China.

**Keywords:** albumin, cardiovascular risk, coronary heart disease, cross-sectional study, diabetes, hypertension, NHANES, RAR, red cell distribution width

## Abstract

While significant evidence has linked various biomarkers to cardiovascular risk, the role of the red cell distribution width to albumin ratio (RAR) in predicting coronary heart disease (CHD) remains underexplored. This cross-sectional study utilized data from 48,928 participants from the National Health and Nutrition Examination Survey (NHANES) 1999 to 2018. The exposure variable was RAR, calculated as the ratio of red cell distribution width to serum albumin levels. CHD was determined based on self-reported data. Multivariate logistic regression was used to examine the relationship between RAR and CHD, adjusting for potential confounders, with stratified analyses by sex, diabetes status, and hypertension status. The mean age of participants was 49.64 ± 18.17 years, with a mean RAR of 3.15 ± 0.51. In the fully adjusted model (Model 3), an increase in RAR was associated with an increased risk of CHD (OR = 1.31, 95% CI: 1.13, 1.51, *P* < .001). Stratified analysis revealed that higher RAR quartiles were associated with higher CHD risk, especially in females and those with diabetes. But no significant interaction was found between RAR and gender or diabetes status on CHD risk. Our study suggests that RAR is associated with an increased risk of CHD, particularly at higher quartiles. While RAR may help clinicians identify individuals at elevated risk for CHD, further longitudinal studies and mechanistic investigations are needed to better understand its predictive value and clinical applicability.

## 1. Introduction

Coronary heart disease (CHD) is a major cause of morbidity and mortality worldwide, characterized by the development of atherosclerosis in the coronary arteries, leading to myocardial infarction and heart failure.^[1,2]^ Epidemiological data demonstrate that CHD continues to be the foremost cause of mortality worldwide, responsible for approximately 7 million deaths and 129 million disability-adjusted life years annually, representing a substantial global health and economic burden.^[3]^The pathogenesis of CHD involves complex interactions between genetic predisposition and environmental factors, such as smoking, high blood pressure, diabetes, and hyperlipidemia.^[4]^ Genetic variants, particularly those related to lipid metabolism and blood pressure regulation, play a significant role in the development of CHD.^[1,5]^ Among the traditional risk factors, dyslipidemia, obesity, and hypertension have been consistently linked to an increased risk of CHD.^[6]^ Additionally, emerging risk factors, including inflammatory markers and insulin resistance, are gaining attention in recent studies as key contributors to CHD.^[7,8]^ These risk factors, can be modifiable, offering opportunities for early detection and prevention strategies. Understanding these mechanisms and identifying potential biomarkers are crucial for the prevention and management of CHD. Furthermore, while current biomarkers such as high-sensitivity C-reactive protein and interleukin-6 reflect inflammation and atherosclerosis, their diagnostic utility is limited by factors such as low specificity and lack of standardization. Therefore, identifying novel biomarkers with greater sensitivity and specificity remains a key area of research.^[9]^

Red blood cell distribution width (RDW), a measure of the variability in red blood cell size, has emerged as an important marker of systemic inflammation, oxidative stress, and endothelial dysfunction.^[10–12]^ Elevated RDW levels have been consistently associated with increased cardiovascular risk, including myocardial infarction, stroke, and mortality, reflecting its involvement in the pathophysiology of atherosclerosis.^[13]^ RDW is considered an indicator of underlying inflammatory activity and vascular damage, both of which may accelerate the progression of atherosclerotic plaques. However, the exact mechanisms by which RDW influences CHD remain unclear, with its clinical utility limited by low diagnostic specificity, lack of standardization.^[14]^ Albumin, a major plasma protein, is essential for maintaining oncotic pressure and transporting various substances in the blood. Low albumin levels are commonly observed in patients with chronic diseases and are associated with chronic inflammation, malnutrition, and endothelial dysfunction.^[15,16]^ Lower albumin levels have been linked to poor cardiovascular outcomes and higher mortality in CHD patients.^[15,17]^ Both RDW and albumin concentrations have been shown to be associated with coronary heart disease, reflecting this relationship from different perspectives. The integration of these two biomarkers may provide a more comprehensive understanding of their association with coronary heart disease, offering deeper insights into systemic inflammation, oxidative stress, and nutritional status. Recently, the red blood cell distribution width to albumin ratio (RAR) has emerged as a simple and promising biomarker. Research has demonstrated its association with poor prognosis in conditions such as diabetes,^[18]^ pulmonary embolism,^[19]^ and acute respiratory failure.^[20]^ Furthermore, a cohort study by Meng Hao^[21]^ further indicated that a higher baseline RAR is linked to an increased risk of all-cause and cause-specific mortality in the general population. However, the relationship between RAR and CHD remains insufficiently investigated in the current literature.

Data from the National Health and Nutrition Examination Survey (NHANES) were used for this cross-sectional analysis. The aim of the study was to investigate the association between the RAR and CHD. It was hypothesized that higher RAR levels would be associated with an increased risk of CHD.

## 2. Methods

### 2.1. Study design and population

This cross-sectional study utilized data from the NHANES to examine the association between the RAR and CHD. NHANES is a nationally representative survey conducted by the National Center for Health Statistics (NCHS), using a complex, multistage probability sampling design to collect health and nutritional data from U.S. adults and children. The Ethics Review Committee of the NCHS approved the NHANES research plan. All the research participants provided written informed consent. More detailed information can be found at https://www.cdc.gov/nchs/nhanes/about/erb.html?CDC_AAref_Val=https://www.cdc.gov/nchs/nhanes/irba98.htm. We used data from the NHANES database collected between 1999 and 2018. These de-identified, publicly available data were accessed on March 19, 2025, from the CDC NHANES website. The authors had no access to information that could identify individual participants.

**Figure 1.**
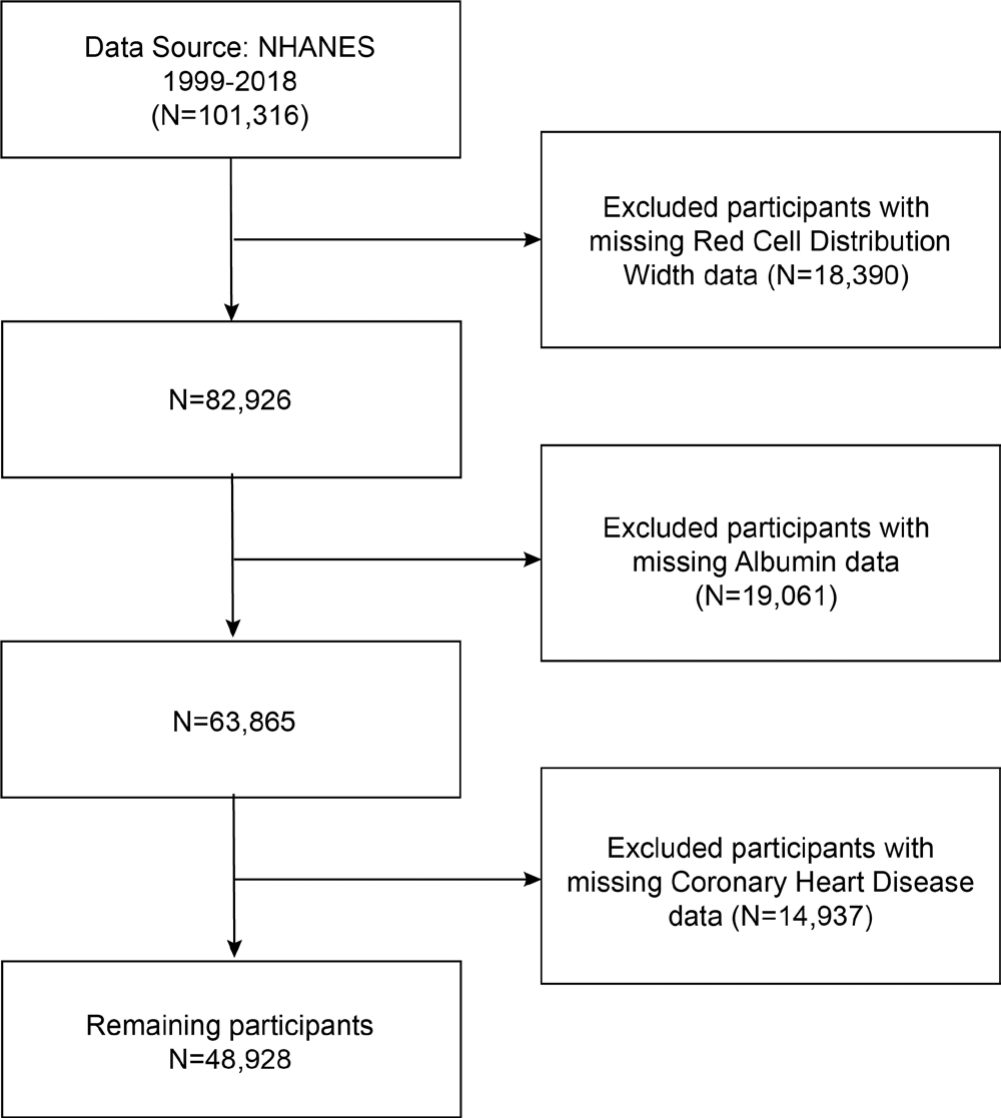
Flowchart of the sample selection from NHANES 1999–2018. NHANES = National Health and Nutrition Examination Survey.

We conducted a cross-sectional study using data from the NHANES spanning 1999 to 2018. A total of 101,316 participants were initially considered. Participants were excluded if they had missing data on RDW (n = 18,390), serum albumin (n = 19,061), or coronary heart disease status (n = 14,937). After applying these exclusion criteria, a final sample of 48,928 participants was included in the analysis (Fig. 1).

### 2.2. Exposure and outcome variables

The primary exposure variable was the RAR, calculated by dividing RDW by serum albumin levels.^[21]^ The outcome variable was CHD, which was diagnosed based on self-reported data. Participants were asked, “Has a doctor or other health professional ever told you that you had coronary heart disease?” and participants who answered “yes” were considered as having CHD.^[22]^

### 2.3. Potential covariates

Covariates in this study included both continuous and categorical variables. The continuous variables were: age (years), poverty income ratio, body mass index (BMI, kg/m²), waist circumference (cm), high-density lipoprotein (HDL, mmol/L), total cholesterol (TC, mmol/L), triglycerides (TG, mmol/L), RDW, and albumin (g/dL).Categorical variables included: gender (male, female), race (Mexican American, other Hispanic, non-Hispanic white, non-Hispanic Black, other race), education level (less than high school, high school or equivalent, above high school), diabetes status (yes, no), and hypertension status (yes, no). Diabetes and hypertension status were determined based on self-reported data, as follows: Participants were asked, “Has a doctor or other health professional ever told you that you have diabetes or sugar diabetes?” Those who answered “yes” were classified as having diabetes, while all other responses were considered as non-diabetic. Similarly, hypertension status was defined based on self-report: participants were asked, “Has a doctor or other health professional ever told you that you have hypertension, also called high blood pressure?” Those who answered “yes” were classified as having hypertension, while all other responses were considered as non-hypertensive.

### 2.4. Statistical analysis

Statistical analyses were performed using R, version 4.3.2 (R Foundation for Statistical Computing, Vienna, Austria), and EmpowerStats (http://www.empowerstats.com, X&Y Solutions, Inc., Boston). The significance level was set at *P* < .05.

Baseline characteristics of the participants were presented as means with 95% confidence intervals (CIs) for continuous variables and proportions for categorical variables. The quartiles of the RAR were compared with Student’s *t* test, the Mann–Whitney *U* test, and the chi-square test for categorical variables. Multivariate logistic regression models were employed to examine the association between RAR and CHD. Model 1 was unadjusted. In Model 2, adjustments were made for gender, age, and race. Model 3 further adjusted for gender, age, race, education level, diabetes status, hypertension status, poverty income ratio, BMI, HDL, TC, and TG. The results from the logistic regression analysis are presented as odds ratios (ORs) and 95% CIs. Moreover, a generalized additive model (GAM) was used to assess the dose-response effect between RAR and CHD. The relationship between RAR and CHD was further explored in the overall population as well as stratified by sex, diabetes status, and hypertension status. The interaction between RAR and sex, diabetes status, and hypertension status was also assessed using interaction tests within the regression models.

## 3. Results

A total of 48,928 participants were included in this study, with 23,608 males (48.25%) and 25,320 females (51.75%). The mean age of the participants was 49.64 ± 18.17 years, and the mean RAR was 3.15 ± 0.51. Table 1 presents the baseline demographic and clinical characteristics of the participants, stratified by quartiles of RAR. Statistical analysis revealed significant differences across the quartiles of RAR for all variables, with the exception of HDL, which had a *P* value of .0867, indicating no statistically significant difference. Participants in the higher RAR quartiles (Q3 and Q4) exhibited higher levels of age, BMI, waist circumference, RDW, and a higher prevalence of diabetes, hypertension, and CHD compared to those in the lower quartiles (Q1 and Q2). In contrast, participants in the higher RAR quartiles demonstrated lower poverty income ratios and albumin levels.

**Table 1:** The demographic and clinical characteristics of the patients by quartiles of baseline RAR.

Variable	RAR Quartiles				
	Q1 (2.08–2.82)	Q2 (2.83–3.04)	Q3 (3.05–3.35)	Q4 (3.36–12.08)	*P* value
Participants (n)	12,117	11,504	12,972	12,335	
Age (yr)	40.68 (40.20, 41.16)	46.95 (46.47, 47.42)	50.34 (49.81, 50.86)	52.34 (51.85, 52.84)	<.0001
Poverty income ratio	3.13 (3.07, 3.19)	3.05 (2.99, 3.11)	2.92 (2.85, 2.98)	2.55 (2.49, 2.61)	<.0001
BMI (kg/m^2^)	26.43 (26.28, 26.57)	28.15 (27.97, 28.32)	29.68 (29.49, 29.87)	32.00 (31.75, 32.25)	<.0001
Waist circumference (cm)	93.00 (92.59, 93.40)	97.40 (96.95, 97.85)	100.66 (100.17, 101.14)	104.92 (104.36, 105.47)	<.0001
HDL (mmol/L)	1.37 (1.36, 1.38)	1.38 (1.36, 1.39)	1.38 (1.36, 1.39)	1.39 (1.38, 1.40)	.0867
TC (mmol/L)	5.11 (5.08, 5.14)	5.14 (5.12, 5.17)	5.07 (5.04, 5.10)	4.93 (4.89, 4.97)	<.0001
TG (mmol/L)	1.68 (1.64, 1.71)	1.74 (1.70, 1.78)	1.71 (1.67, 1.75)	1.66 (1.62, 1.70)	.0162
Red cell distribution width (%)	12.20 (12.18, 12.21)	12.71 (12.69, 12.73)	13.16 (13.14, 13.18)	14.55 (14.51, 14.60)	<.0001
Albumin (g/dL)	4.60 (4.59, 4.60)	4.34 (4.33, 4.34)	4.14 (4.14, 4.15)	3.87 (3.86, 3.87)	<.0001
Gender (%)					<.0001
Male	61.92 (61.04, 62.80)	51.35 (50.09, 52.60)	42.22 (41.05, 43.39)	30.64 (29.48, 31.83)	
Female	38.08 (37.20, 38.96)	48.65 (47.40, 49.91)	57.78 (56.61, 58.95)	69.36 (68.17, 70.52)	
Race (%)					<.0001
Mexican American	7.98 (6.95, 9.14)	8.52 (7.38, 9.82)	8.07 (6.93, 9.39)	8.22 (7.01, 9.61)	
Other Hispanic	5.28 (4.33, 6.42)	5.74 (4.79, 6.86)	5.76 (4.80, 6.89)	6.06 (5.17, 7.10)	
Non-Hispanic white	74.91 (73.01, 76.73)	70.68 (68.45, 72.81)	67.85 (65.30, 70.29)	57.03 (54.17, 59.85)	
Non-Hispanic Black	4.72 (4.17, 5.35)	8.11 (7.17, 9.18)	11.94 (10.68, 13.34)	21.58 (19.36, 23.97)	
Other race	7.11 (6.35, 7.96)	6.95 (6.19, 7.79)	6.38 (5.72, 7.11)	7.11 (6.29, 8.02)	
Education (%)					<.0001
Less than high school	4.73 (4.27, 5.25)	5.92 (5.36, 6.54)	6.49 (5.92, 7.11)	7.72 (7.09, 8.39)	
High school or equivalent	32.77 (31.14, 34.44)	34.52 (32.87, 36.20)	35.52 (34.13, 36.94)	39.56 (38.17, 40.98)	
Above high school	62.50 (60.69, 64.27)	59.56 (57.65, 61.43)	57.99 (56.37, 59.59)	52.72 (51.11, 54.32)	
Diabetes (%)	3.89 (3.49, 4.34)	6.93 (6.37, 7.54)	10.53 (9.85, 11.26)	16.55 (15.72, 17.41)	<.0001
Hypertension (%)	20.49 (19.46, 21.56)	28.07 (26.92, 29.25)	34.34 (33.03, 35.67)	43.02 (41.74, 44.31)	<.0001
Coronary heart disease (%)	1.77 (1.52, 2.06)	2.52 (2.19, 2.90)	4.35 (3.87, 4.88)	6.18 (5.57, 6.86)	<.0001

BMI = body mass index, HDL = high-density lipoprotein, RAR = red cell distribution width to albumin ratio, TC = total cholesterol, TG = triglycerides.

**Figure 2.**
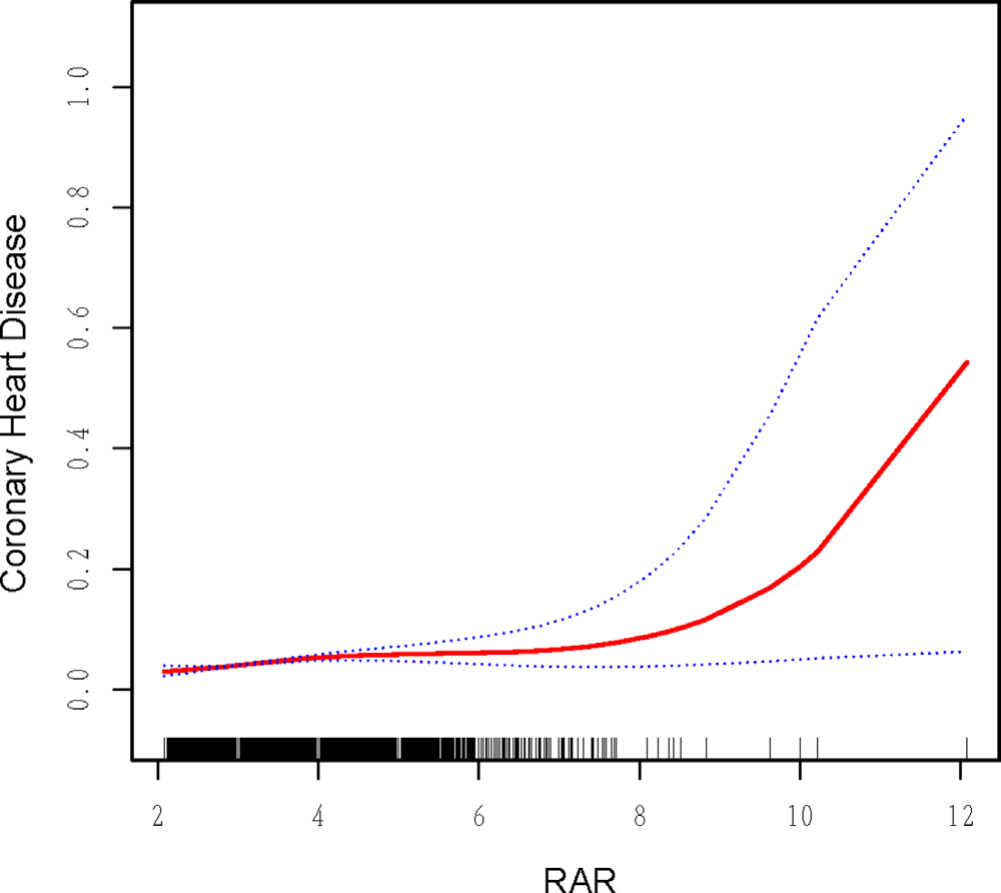
Association between RAR and CHD. A linear association between RAR and the prevalence of CHD. The solid line and dashed line represent the estimated values and their corresponding 95% confidence interval. Adjustment factors included gender, age, race, education level, diabetes status, hypertension status, poverty income ratio, BMI, waist circumference, HDL, TC, and TG. BMI = body mass index, CHD = coronary heart disease, HDL = high-density lipoprotein, RAR = red cell distribution width to albumin ratio, TC = total cholesterol, TG = triglycerides.

Multivariate logistic regression analysis showed a significant association between RAR and CHD across all models (Table 2). In Model 1, the unadjusted OR for CHD per unit increase in RAR was 1.97 (*P* < .001). After adjusting for gender, age, and race in Model 2, the OR decreased to 1.64 (*P* < .001). In Model 3, which further adjusted for education level, diabetes status, hypertension status, poverty income ratio, BMI, waist circumference, HDL, TC, and TG, the OR decreased to 1.31 (*P* < .001), indicating that RAR remained associated with CHD even after accounting for multiple confounders. The results from the multivariate regression analysis were consistent with the fitting curve presented in Figure 2, which shows a positive dose-response relationship between RAR and CHD risk.

**Table 2:** Relative odds of coronary heart disease according to RAR in different models among all participants.

	Coronary heart disease OR (95% CI)		
	Model 1	Model 2	Model 3
RAR	1.97 (1.83, 2.12)***	1.64 (1.48, 1.82)***	1.31 (1.13, 1.51)***
RAR quartile			
Q1 (2.08–2.82)	Reference	Reference	Reference
Q2 (2.83–3.04)	1.44 (1.18, 1.75)***	0.96 (0.78, 1.18)	0.89 (0.72, 1.11)
Q3 (3.05–3.35)	2.52 (2.09, 3.05)***	1.38 (1.12, 1.69)**	1.16 (0.94, 1.43)
Q4 (3.36–12.08)	3.66 (3.06, 4.37)***	1.87 (1.53, 2.29)***	1.33 (1.08, 1.64)**
*P* for trend	<.0001	<.0001	.0008

Model 1 was adjusted for none.

Model 2 was adjusted for gender, age, and race.

Model 3 was adjusted for gender, age, race, education level, diabetes status, hypertension status, poverty income ratio, BMI, waist circumference, HDL, TC, and TG.

BMI = body mass index, HDL = high-density lipoprotein, RAR = red cell distribution width to albumin ratio, TC = total cholesterol, TG = triglycerides.

** *P* < .01.

*** *P* < .001.

For the quartiles of RAR, participants in the higher quartiles (Q3 and Q4) showed a higher risk of CHD in all models. In Model 1, the OR for Q3 was 2.52 (95% CI: 2.09, 3.05, *P* < .001) and for Q4 was 3.66 (95% CI: 3.06, 4.37, *P* < .001). In Model 2, the ORs for Q3 and Q4 decreased to 1.38 (95% CI: 1.12, 1.69, *P* = .0025) and 1.87 (95% CI: 1.53, 2.29, *P* < .0001), respectively, but remained statistically significant. In Model 3, the OR for Q3 was 1.16 (95% CI: 0.94, 1.43) and for Q4 was 1.33 (95% CI: 1.08, 1.64), which were still significant, indicating an association between higher RAR and increased CHD risk. With a *P* for trend < .001, indicating a linear positive correlation between RAR and CHD.

Table 3 presents the association between RAR and CHD stratified by gender. In males, the relationship between RAR and CHD approached borderline statistical significance (OR = 1.17, *P* < .05), suggesting a mild association. In contrast, in females, the association was more pronounced and statistically significant (OR = 1.49, *P* < .001). The *P* for interaction was .0588, indicating no significant interaction between gender and RAR on CHD risk. Regarding RAR quartiles, in males, no significant differences were observed across quartiles. In females, the highest quartiles (Q4 OR = 1.64, *P* < .05) was associated with increased CHD risk. Additionally, the *P* for trend in females was .0008, indicating a strong linear association between RAR and CHD risk.

**Table 3:** Relative odds of coronary heart disease according to RAR in adjusted model among male and female participants.

	Male		Female		
	Events (%)	Coronary heart disease OR (95% CI)	Events (%)	Coronary heart disease OR (95% CI)	*P* value for interaction
RAR	23,608	1.17 (1.01, 1.36)*	25,320	1.49 (1.22, 1.82)***	.0588
RAR quartile					.5082
Q1 (2.08–2.82)	7649 (32.38)	Reference	4468 (17.64)	Reference	
Q2 (2.83–3.04)	6114 (25.89)	0.87 (0.67, 1.12)	5390 (21.30)	1.00 (0.68, 1.46)	
Q3 (3.05–3.35)	5725 (24.23)	1.06 (0.81, 1.38)	7247 (28.64)	1.47 (1.00, 2.15)	
Q4 (3.36–12.08)	4120 (17.44)	1.22 (0.95, 1.57)	8215 (32.46)	1.64 (1.13, 2.40)*	
*P* for trend		.0566		.0008	

This model was adjusted for age, race, education level, diabetes status, hypertension status, poverty income ratio, BMI, waist circumference, HDL, TC, and TG.

BMI = body mass index, HDL = high-density lipoprotein, RAR = red cell distribution width to albumin ratio, TC = total cholesterol, TG = triglycerides.

* *P* < .05.

*** *P* < .001.

Table 4 presents the association between RAR and CHD stratified by diabetes status. In the diabetic group, RAR was significantly associated with an increased risk of CHD. However, the *P* for interaction was .4513, indicating no significant interaction between diabetes status and RAR on CHD risk. When evaluating RAR quartiles, a positive association was observed only in the diabetic group, with participants in the Q4 quartile showing an OR of 1.64 (*P* < .05). The *P* for trend in the diabetic group was .0048, suggesting a significant linear relationship between RAR and CHD risk.

**Table 4:** Associations between RAR and coronary heart disease in adjusted models among non-diabetic and diabetic participants.

	Non-diabetic		Diabetic		
	Events (%)	Coronary heart disease OR (95% CI)	Events (%)	Coronary heart disease OR (95% CI)	*P* value for interaction
RAR	43,038	1.19 (0.96, 1.46)	5890	1.32 (1.10, 1.57)**	.4513
RAR quartile					.4659
Q1 (2.08–2.82)	11,403 (26.50)	Reference	714 (12.12)	Reference	
Q2 (2.83–3.04)	10,440 (24.26)	0.84 (0.68, 1.05)	1064 (18.06)	1.05 (0.61, 1.83)	
Q3 (3.05–3.35)	11,264 (26.17)	1.04 (0.83, 1.31)	1708 (28.00)	1.44 (0.89, 2.34)	
Q4 (3.36–12.08)	9931 (23.07)	1.11 (0.89, 1.39)	2404 (40.81)	1.64 (1.01, 2.66)*	
*P* for trend		.1347		.0048	

This model was adjusted for gender, age, race, education level, hypertension status, poverty income ratio, BMI, waist circumference, HDL, TC, and TG.

BMI = body mass index, HDL = high-density lipoprotein, RAR = red cell distribution width to albumin ratio, TC = total cholesterol, TG = triglycerides.

* *P* < .05.

** *P* < .01.

Table 5 presents the association between RAR and CHD stratified by hypertension status. In the hypertensive group, RAR was associated with an increased risk of CHD. However, the *P* for interaction was .6852, indicating no interaction between hypertension status and RAR on CHD risk. When evaluating RAR quartiles, although the ORs for Q3 and Q4 were greater than 1, neither reached statistical significance.

**Table 5:** Associations between RAR and coronary heart disease in adjusted models among non-hypertensive and hypertensive participants.

	Non-hypertensive		Hypertensive		
	Events (%)	Coronary heart disease OR (95% CI)	Events (%)	Coronary heart disease OR (95% CI)	*P* value for interaction
RAR	32,042	1.18 (0.97, 1.44)	16,886	1.25 (1.04, 1.49)*	.6852
RAR quartile					.8877
Q1 (2.08–2.82)	9397 (29.31)	Reference	2720 (16.12)	Reference	
Q2 (2.83–3.04)	7934 (24.80)	0.83 (0.57, 1.23)	3570 (21.14)	0.89 (0.68, 1.16)	
Q3 (3.05–3.35)	7983 (24.94)	1.18 (0.81, 1.74)	4989 (29.54)	1.07 (0.83, 1.38)	
Q4(3.36–12.08)	6728 (21.00)	1.17 (0.81, 1.68)	5607 (33.21)	1.22 (0.95, 1.57)	
*P* for trend		.1545		.0244	

This model was adjusted for gender, age, race, education level, diabetes status, poverty income ratio, BMI, waist circumference, HDL, TC, and TG.

BMI = body mass index, HDL = high-density lipoprotein, RAR = red cell distribution width to albumin ratio, TC = total cholesterol, TG = triglycerides.

* *P* < .05.

## 4. Discussion

This large-scale cross-sectional study, based on data from the 1999 to 2018 NHANES, aimed to investigate the association between RAR and CHD. The analysis demonstrated a significant positive association between RAR levels and the risk of CHD in all participants. Although the OR decreased after adjusting for confounding factors, a positive association remained, particularly among individuals with higher RAR levels. Stratified analysis further revealed that this association was most pronounced in females and those with diabetes, with higher RAR quartiles being linked to increased CHD risk.

Previous studies have shown that both RDW and albumin, as components of the RAR, are associated with adverse cardiovascular outcomes. Elevated RDW has been linked to increased risks of cardiovascular events, including myocardial infarction and stroke.^[13,23,24]^ Similarly, low albumin levels have been identified as predictive of poor cardiovascular outcomes, often reflecting chronic conditions such as heart failure and chronic kidney disease.^[15,25,26]^ In our study, the introduction of RAR revealed a significant positive correlation with CHD, particularly in individuals with higher RAR quartiles. This is consistent with findings from other studies. For instance, Huang et al^[27]^ observed a significant association between RAR and carotid plaque in CHD patients, and with a stronger correlation observed in females, mirroring our subgroup analysis. Furthermore, Chen et al^[28]^ found that high RAR levels were linked to increased 30-day, 90-day, and 1-year mortality in ICU patients with CHD and diabetes, highlighting RAR’s potential as a tool to identify individuals at elevated risk for adverse outcomes. Additionally, RAR has been associated with poor prognoses in various other conditions, including diabetes,^[18]^ stroke,^[29]^ pancreatitis,^[30]^ pulmonary embolism,^[19]^ and aortic aneurysm.^[31]^ Hao et al^[21]^ demonstrated in a large cohort study that higher baseline RAR was linked to an increased risk of all-cause mortality, as well as specific causes of death such as cancer, heart disease, cerebrovascular disease, respiratory diseases, Alzheimer’s disease, and diabetes. RAR is derived from RDW and albumin, both of which are widely available laboratory indicators, enabling more comprehensive assessments of patient health and prognosis.

The mechanisms underlying the association between RAR and CHD are not fully understood; however, several potential biological mechanisms can be considered. RDW reflects the heterogeneity of red blood cell volume and is associated with increased inflammation and oxidative stress, both of which are key contributors to the development of cardiovascular diseases.^[32–34]^ Hypoalbuminemia, on the other hand, is considered a marker of systemic inflammation, malnutrition, and endothelial dysfunction, all of which may elevate cardiovascular risk.^[17,35,36]^ RAR may amplify these changes, reflecting endothelial dysfunction and arterial stiffness, which are crucial in the progression of CHD. Previous studies have showed that higher RAR is associated with an increased risk of peripheral arterial disease.^[37]^ Additionally, a study has shown that RAR is significantly associated with carotid plaques in CHD patients.^[27]^ Moreover, hyperglycemia and insulin resistance exacerbate inflammation and oxidative stress, while simultaneously reducing albumin synthesis, further increasing RAR and accelerating coronary artery calcification.^[38,39]^ These findings align with our results, which show that RAR is more sensitive to CHD risk in diabetic patients compared to non-diabetic individuals. Other studies have suggested that RAR may reflect immune dysregulation, which could potentially contribute to the development of CHD.^[40,41]^ However, the exact mechanisms through which RAR influences CHD remain to be fully elucidated, and further research is needed to explore their relationship with CHD and to validate the clinical utility of RAR in risk prediction and stratification.

### 4.1. Limitations and strengths

A key strength of our study is the large sample size, which enhances the representativeness of the findings and supports their generalizability to diverse populations. However, as a cross-sectional study, the design limits our ability to establish causal relationships between RAR and CHD. While we adjusted for a wide range of potential confounders, there may still be residual confounding that could influence the observed associations. Additionally, as RAR is a composite measure based on RDW and albumin, the interpretation of RAR is complex and requires careful consideration of the individual contributions of these 2 markers. We also acknowledge 2 additional limitations: the time span of data collection (1999–2018) may have introduced inconsistencies due to medical and technological advances over the years, which could have affected data accuracy and consistency. Furthermore, diagnoses such as CHD, diabetes, and hypertension were based on self-reported data rather than actual medical diagnoses, which could introduce inaccuracies or underreporting, potentially influenced by sociodemographic and other factors.

## 5. Conclusions

In conclusion, our study suggests that higher RAR is associated with an increased risk of CHD, particularly in women and individuals with diabetes. Further studies are required to explore the underlying mechanisms linking RAR to cardiovascular diseases and its potential in assessing long-term cardiovascular outcomes. Longitudinal studies, including genetic investigations and clinical trials, are essential for validating RAR’s utility in cardiovascular risk stratification and management.

## Acknowledgments

We thank the participants of the NHANES and the NCHS for providing the data used in this study.

## Author contributions

**Conceptualization:** Rong Lei, Huiling Liang, Xumeng Ding, Chaofu Yue, Mei Yang.

**Data curation:** Rong Lei, Huiling Liang, Xumeng Ding, Chaofu Yue, Xian Huang, Qiaolin Li.

**Formal analysis:** Rong Lei, Huiling Liang, Xumeng Ding, Qiaolin Li, Mei Yang.

**Funding acquisition:** Mei Yang.

**Investigation:** Rong Lei, Huiling Liang, Chaofu Yue, Wei Bao.

**Methodology:** Rong Lei, Xumeng Ding, Xian Huang, Wei Bao, Qi Qiu.

**Project administration:** Rong Lei, Huiling Liang, Xumeng Ding.

**Resources:** Chaofu Yue, Xian Huang.

**Software:** Rong Lei, Chaofu Yue, Xian Huang, Qi Qiu.

**Supervision:** Huiling Liang, Qiaolin Li, Mei Yang.

**Validation:** Rong Lei, Huiling Liang, Xumeng Ding, Xian Huang, Wei Bao, Qi Qiu.

**Visualization:** Rong Lei, Xumeng Ding, Xian Huang, Qiaolin Li.

**Writing – original draft:** Rong Lei, Huiling Liang, Xumeng Ding, Mei Yang.

**Writing – review & editing:** Chaofu Yue, Xian Huang, Qiaolin Li, Wei Bao, Qi Qiu.
